# Chemical Characterization and In Vitro Biological Activities of Essential Oil from *Oxalis corniculata* L. Collected in the Ecuador Highlands

**DOI:** 10.3390/plants15193047

**Published:** 2026-10-06

**Authors:** Christian Miguel Moncayo Rivera, Lourdes Inés Pascual, Carlos Gamarra-Luques

**Affiliations:** 1 Instituto de Fisiología, Facultad de Ciencias Médicas (FCM), Universidad Nacional de Cuyo, Centro Universitario, Mendoza M5502JJMA, Argentina; christianmoncayo487@gmail.com; 2 Instituto de Medicina y Biología Experimental de Cuyo (IMBECU), Universidad Nacional de Cuyo, CCT Mendoza CONICET, Ruiz Leal s/n, Mendoza M5502JJMA, Argentina; lourdes.p.97@gmail.com; 3 Facultad de Ciencias Exactas y Naturales, Universidad Nacional de Cuyo, Padre Jorge Contreras 1300, Parque General San Martín, Mendoza M5502JMA, Argentina

**Keywords:** Oxalidaceae, native plants, antioxidants, antimicrobial, cytotoxicity

## Abstract

*Oxalis corniculata* L. is an ethnobotanically recognized plant in Ecuador. Although its bioactive extracts have been investigated, the chemical and biological properties of its essential oil (OcEO) remain unexplored. This study aimed to characterize the chemical profile of OcEO and evaluate its in vitro antioxidant, antimicrobial, and cytotoxic properties. Aerial parts of *O. corniculata* were collected and hydrodistilled to obtain the OcEO. Its phytochemical profile was characterized using GC-MS and GC-FID. Antioxidant capacity was measured via DPPH, ABTS, and FRAP assays. Antimicrobial activity against *Klebsiella pneumoniae*, *Acinetobacter baumannii*, and *Candida albicans* was evaluated by determining the minimum inhibitory concentration (MIC) and zone of inhibition (ZOI). Cytotoxicity was assessed on MCF-7, HCT-116, and MRC-5 cell lines using the MTT assay, determining the IC_50_ and Selective Cytotoxicity Index (SCI). The GC-MS and GC-FID analysis identified or tentatively identified 24 constituents (accounting for 93.2% of the total oil composition), predominantly sesquiterpenes (54.96%), with β-caryophyllene (24.71%) and germacrene D (14.62%) as the major components. OcEO showed moderate complementary chemical antioxidant and antimicrobial effects, being most active against *K. pneumoniae* (MIC: 0.56 mg/mL) and *C. albicans* (MIC: 2.24 mg/mL). Furthermore, OcEO evidenced selective cytotoxicity against tumor cells, particularly against HCT-116 (IC_50_: 8.19 mg/mL; SCI: 4.52). In conclusion, OcEO exhibits potential in vitro antioxidant, antimicrobial, and cytotoxic properties, providing preliminary evidence to support future research on this Ecuadorian essential oil.

## 1. Introduction

The search for new plant-derived bioactive molecules remains a key strategy for developing therapeutic, nutraceutical, and phytopharmaceutical agents [1]. Despite significant advances in chemical synthesis, a considerable proportion of currently used drugs are derived directly or indirectly from natural products [2]. This highlights the value of biodiversity as a source of compounds suitable for biological screening to identify plant-derived bioactive constituents [3]. In this context, megadiverse countries such as Ecuador represent exceptional reservoirs of plant species with medicinal potential that remain insufficiently explored from a phytochemical and pharmacological perspective [4].

*Oxalis corniculata* L. (Oxalidaceae), commonly known as “chulco”, “platanitos”, “agritos”, “vinagrillo”, “vinagrillo rastrero”, “acederilla”, creeping wood sorrel or procumbent yellow sorrel, is an herbaceous species widely distributed in tropical, subtropical, and temperate regions worldwide. Although its geographic origin was debated for decades, archaeobotanical, historical, and ethnobotanical evidence indicates that it likely originated in Southeast Asia, from where it spread early to other continents, becoming one of the most widely distributed plant species worldwide [5].

The extensive distribution of *O. corniculata* has facilitated its incorporation into numerous traditional medicine systems. Various communities in Asia, Africa, and the Americas use it to treat a range of ailments, demonstrating a long-standing association between this species and human health [5,6]. In Ecuador, this plant is part of the ethnobotanical heritage and is traditionally used to treat fever, bilious disorders, kidney diseases, and intestinal parasites [7].

In recent years, the growing interest in *O. corniculata* has prompted numerous phytochemical investigations that have identified a remarkable diversity of secondary metabolites. These include flavonoids, phenolic acids, tannins, terpenoids, alkaloids, polysaccharides, and other phenolic compounds, which are responsible for multiple biological activities. Notably, its essential oil represents a poorly studied chemical fraction of special interest due to the presence of monoterpenes and volatile compounds associated with antioxidant, antimicrobial, and anti-inflammatory properties [8].

Pharmacological research conducted with aqueous, alcoholic and organic extracts of *O. corniculata* has reported a wide range of biological activities, including antioxidant, antimicrobial, anti-inflammatory, hypoglycemic, hepatoprotective, neuroprotective, and antitumor effects. Several in vitro studies have demonstrated high free radical-scavenging capacity, significant inhibition of pathogenic bacterial growth, and cytotoxic effects against different tumor cell lines. These findings support the potential of *O. corniculata* as a source of bioactive compounds for pharmaceutical and biomedical research [6,8,9].

However, despite the growing number of publications on this species, the chemical composition of the *O. corniculata* essential oil (OcEO) from Ecuadorian populations remains poorly characterized. Consequently, its biological properties remain largely unexplored. Considering the high chemical variability of secondary metabolites associated with geographic origin, environmental conditions, and genetic factors, characterizing the local chemical profile is essential for understanding the medicinal potential and biotechnological applications of this essential oil.

Given that in vitro results do not account for limitations related to bioavailability, dose-dependent toxicity, or findings from clinical trials, subsequent in vivo validation is required [10]. Therefore, the aim of this study was to characterize the chemical profile of OcEO from the Ecuadorian highlands and evaluate its in vitro antioxidant, antimicrobial, and cytotoxic properties, providing scientific evidence to support further research on this Ecuadorian species.

## 2. Results and Discussion

### 2.1. OcEO Characterization and Composition

The essential oil yield was 0.393 ± 0.001% (393 ± 1 mg/100 g of fresh material) based on three independent hydrodestillations, with a measured density of 0.894 ± 0.03 g/mL. The essential oil was analyzed to identify and quantify its constituents using gas chromatography–mass spectrometry (GC-MS) and gas chromatography coupled with a flame ionization detector (GC-FID), respectively. This analysis led to the confirmed identification of 17 phytochemicals, while 7 were tentatively identified, accounting for 93.2% of the total oil composition. The total ion gas chromatogram of OcEO obtained on a DB-5ms column is shown in Figure 1, and its constituents are listed in Table 1, in elution order. Among them, the main compounds detected were β-caryophyllene (24.71 ± 0.71%), germacrene D (14.62 ± 0.39%), limonene (8.36 ± 0.24%), caryophyllene oxide (5.41 ± 0.14%), and α- and β-pinene (8.67 ± 0.17%).

The main fraction of the essential oil consisted of sesquiterpene hydrocarbons (44.28%), including β-caryophyllene, α-humulene, germacrene D, and β-farnesene (peaks 15–18). This was followed by monoterpene hydrocarbons (21.4%), including α-pinene, sabinene, β-pinene, myrcene, *p*-cymene, limonene, and γ-terpinene (peaks 2–7, 9); oxygenated sesquiterpenes (10.68%), including nerolidol, spathulenol and caryophyllene oxide (peaks 19–21); oxygenated monoterpenes (5.3%), including 1,8-cineole, terpinen-4-ol, and α-terpineol (peaks 8, 11, 12); and phenolic monoterpenes (3.08%), including thymol and carvacrol (peaks 13, 14). Other minor groups present included the aldehyde hexanal (peak 1); the ketone fenchone (peak 10); the fatty acid hexadecanoic acid (peak 22); the diterpene alcohol phytol (peak 23); and the triterpene hydrocarbon squalene (peak 24).

To our knowledge, only one previous study has characterized the essential oil obtained from the leaves of *O. corniculata*. The plant specimens for that study were collected in Zhaoqing City, Guangdong Province, China, and the extraction was performed using supercritical CO_2_ [11].

The comparison of the two phytochemical profiles reveals marked differences. In our study, the oil obtained from the aerial parts by hydrodistillation was clearly dominated by sesquiterpene and monoterpene hydrocarbons. In contrast, the oil studied in China showed a predominance of monoterpenes and lipids, with geranyl acetate (13.3%), terpinolene (9.2%), hexadecanoic acid (7.7%), linalool oxide (7.4%), and geraniol (6.4%) as the major compounds. Notably, hexadecanoic acid (palmitic acid), which was reported as a major constituent in the Chinese oil, showed a tentative relative abundance of only 1.72% in the Ecuadorian samples. Additionally, both studies reported the presence of several minor constituents, such as β-pinene, myrcene, and terpinen-4-ol.

Although differences in extraction methods may have contributed to the observed variation, the pronounced differences in chemical composition may also reflect the distinct ecological characteristics of the sampling sites. While the Chinese population, occurring at a lower altitude, was characterized by a higher abundance of oxygenated compounds and lipid derivatives, the Ecuadorian population was sampled at a high altitude (3170 m a.s.l.), which may contribute to differences in its chemical profile. This highland environment, characterized by low temperatures, physiological water stress, and elevated ultraviolet (UV-B) radiation, has been associated with the upregulation of biosynthetic pathways of sesquiterpene and monoterpene hydrocarbons as an adaptive photoprotective and antioxidative mechanism against oxidative stress [12,13]. Although further analyses of samples from multiple collections and locations are necessary to confirm the Ecuadorian chemotype and its compositional variability, the results suggest that, potentially, the Ecuadorian population represents a distinct high-altitude chemical profile rich in sesquiterpene and monoterpene hydrocarbons [14]. This metabolic divergence may influence the biological activities of OcEO [15].

### 2.2. OcEO Antioxidant Activity Determination

The in vitro antioxidant activity of OcEO was evaluated by determining its free radical-scavenging capacity using the DPPH and ABTS assays, as well as its ferric-reducing antioxidant power (FRAP). The results, expressed in milligrams of Trolox equivalents per gram of fresh material (mg TE/g), are summarized in Table 2.

The antioxidant activity observed in this study is consistent with previous reports describing *O. corniculata* as a promising source of natural compounds capable of mitigating oxidative stress [16,17,18]. Our results fall within the ranges reported for ethanolic and methanolic extracts of *O. corniculata* collected from various geographical regions, in which phenolic compounds, flavonoids, and antioxidant vitamins (vitamins C and E) have been described as major constituents [16,17].

Interest in plant-based antioxidants, particularly essential oils, has grown due to concerns regarding the potential side effects associated with synthetic antioxidants such as BHT and BHA [17,19]. Essential oils may exert antioxidant effects through two distinct mechanisms: first, by directly neutralizing free radicals; and second, by modulating the activity of endogenous antioxidant enzymes, thereby indirectly supporting cellular defense [20]. The literature extensively reports the ability of essential oils to neutralize reactive oxygen species (ROS) through electron- or hydrogen-atom-transfer mechanisms, as well as through transition-metal chelation. However, the reported antioxidant capacity of essential oils varies widely, and differences in extraction procedures, chemical composition, assay conditions, and endpoint definitions should be considered before drawing conclusions about their relative potency. For instance, *Pistacia lentiscus* essential oil exhibits low free radical-scavenging capacity (DPPH: 0.06 mg TE/g; ABTS: 0.09 mg TE/g) and weak reducing power (FRAP: 6.4 mg TE/g) [17]. In contrast, species from the *Myrtaceae* family, such as *Pimenta racemosa*, exhibit high antioxidant activity (DPPH: 1032.83 mg TE/g; FRAP: 1506.62 mg TE/g), which has been associated with their high eugenol content (70.87%) [21]. Comparatively, the values obtained for OcEO in this study are within the ranges reported for the essential oils of *Boswellia serrata* (DPPH: 9.24 mg TE/g; ABTS: 25.71 mg TE/g; and FRAP: 13.22 mg TE/g) [22], *Curcuma longa* (turmeric) (DPPH: 3.63 mg TE/g; ABTS: 17.58 mg TE/g; and FRAP: 34.75 mg TE/g) [23], and *Origanum vulgare* (DPPH: 0.45 mg TE/g; ABTS: 9.57 mg TE/g; and FRAP: 7.33 mg TE/g) [24].

Although the activities of isolated compounds were not studied in this work, the antioxidant activity observed for OcEO may be related to its characterized phytochemical profile, which is predominantly composed of sesquiterpenes and monoterpenes. Studies on isolated compounds have documented the ability of *β*-caryophyllene to neutralize ROS and inhibit lipid peroxidation [25,26]. Moreover, *β*-caryophyllene has been reported to exhibit synergistic antiradical activity when combined with germacrene D [27]. In this regard, several authors have demonstrated that essential oils rich in β-caryophyllene and germacrene D exhibit synergistic antioxidant capacity due to the reactivity of their endo- and exocyclic double bonds [28]. Additionally, isolated monoterpenes including α-pinene, β-pinene and limonene have been reported to significantly contribute to ferric-reducing activity in the FRAP assay and to the scavenging of the ABTS radical cation [29,30]. Altogether, the concomitant presence of these volatile terpenoids may contribute to the overall complementary chemical antioxidant capacity of OcEO by enhancing its free-radical scavenging activity in vitro, supporting further research to evaluate its effects in cellular and in vivo models.

### 2.3. OcEO Antimicrobial Activity Determination

The findings of this study demonstrate that OcEO possesses antimicrobial activity against both Gram-negative bacilli and opportunistic yeasts (Table 3). Among the Gram-negative bacteria evaluated, *K. pneumoniae* showed greater susceptibility to treatment than *A. baumannii*. In contrast, *C. albicans* showed an intermediate minimum inhibitory concentration (MIC) but the largest zone of inhibition (ZOI). Regarding the positive controls, ciprofloxacin showed significant antimicrobial activity against *K. pneumoniae* and *A. baumanii*, with significantly greater activity than OcEO based on the MIC and ZOI values (Student’s *t* test, *p* < 0.05). Regarding ketoconazole, its antimicrobial activity against *C. albicans* was significantly higher than that of OcEO (Student’s *t* test, *p* < 0.05).

To the best of our knowledge, this is the first report on the antimicrobial activity of OcEO.

Although there is documented evidence of the antimicrobial activity of *O. corniculata* extracts, previous studies have attributed this biological activity to phenolic compounds [9,31,32,33,34]. Among these studies, only Ullah et al. [9] reported activity against the pathogens evaluated in our study. In that study, the ethanolic extract, mainly composed of tannins and flavonoids, exhibited weak activity against *K. pneumoniae*.

Considering the literature on essential oils from other plant species and the previously mentioned limitations on its comparison, the OcEO obtained in our study showed lower antimicrobial activity against *K. pneumoniae* (ATCC 13883) (MIC: 0.56 mg/mL) than that reported for *Origanum vulgare* (MIC: 0.128–0.256 mg/mL) [35]. However, OcEO showed greater antimicrobial activity than the essential oils of *O. compactum* (MIC: 6–10.5 mg/mL), *Thymus serpyllum* (MIC: 17.2–43 mg/mL), and *O. majorana* (MIC: 11–39 mg/mL) [36]. Regarding its activity against *A. baumannii* (ATCC 19606), OcEO showed moderate antimicrobial (MIC: 4.47 mg/mL), falling between the MIC values reported for the essential oils of *Lavandula pubescens* (MIC: 0.078 mg/mL) and *Salvia hypargeia* (MIC: 28.8 mg/mL) [37,38]. Regarding the activity against *C. albicans* (ATCC 10231), our results (MIC: 2.24 mg/mL) showed lower antimicrobial activity than that reported for the essential oils of *Cinnamomun zeylanicum* (MIC: 1–1.25 mg/mL) and *Eugenia caryophyllus* (MIC: 0.75–0.94 mg/mL), in both of which β-caryophyllene was reported as the second most abundant constituent [39].

Although the antimicrobial mechanisms of action were not investigated in this study, the bioactivity of OcEO may be hypothetically associated with its characteristic phytochemical profile, which is dominated by bicyclic sesquiterpenes and monoterpene hydrocarbons. While the major components β-caryophyllene, germacrene D, and caryophyllene oxide, as isolated compounds, are recognized for their role in disrupting the cytoplasmic membrane and promoting membrane leakage [40,41], the monoterpenes, such as limonene, α-pinene and β-pinene, are proposed to facilitate the initial permeabilization of the lipopolysaccharide (LPS)-containing outer membrane in Gram-negative bacteria, allowing the sesquiterpene fraction to reach the inner lipid bilayer [42,43].

In summary, the data suggest that OcEO exhibits in vitro antimicrobial activity, particularly against *K. pneumoniae* and *C. albicans*. Compared with previously reported data, the variability observed in antimicrobial activity underscores the influence of the chemical profile of OcEO and highlights the need for further studies to evaluate the efficacy of essential oils enriched with these phytochemicals.

### 2.4. OcEO Cytotoxic Activity and Selectivity Index Determination

The evaluation of cellular metabolic activity after 48 h of OcEO treatment, using a dose–response design and the MTT assay, demonstrated a differential cytotoxic effect depending on the cell type (Figure 2). The complete dose–response data for each cell line are presented in Table 4.

The HCT-116 colorectal adenocarcinoma cell line exhibited the greatest sensitivity to treatment (IC_50_: 8.19 mg/mL), followed by MCF-7 breast adenocarcinoma cells (IC_50_: 14.59 mg/mL). In contrast, the normal human fibroblast cell line MRC5 showed markedly lower sensitivity (IC_50_: 37.04 mg/mL) (Figure 2a–c). The cytotoxic activity, as indicated by the IC_50_ values, was significantly higher in tumor cell lines than in the non-tumor cell line, suggesting selective cytotoxicity (ANOVA, Dunnett’s post hoc test, *p* < 0.0001) (Figure 2d). The Selective Cytotoxicity Index (SCI) further indicated preferential cytotoxicity of OcEO toward tumor cells, with SCI values of 4.52 for HCT-116 and 2.54 for MCF-7. Although the essential oil exhibited relatively low baseline cytotoxicity, both SCI values exceeded the threshold of 2 reported in the literature [44], supporting further investigation of OcEO selective cytotoxic effects.

Among previous reports on the cytotoxicity of *O. corniculata*, Gholipour et al. [45] evaluated the ethanolic extract of its aerial parts against MCF-7 cells and human dermal fibroblasts (HDF). In that study, cytotoxic activity was attributed to the high concentration of phenolic compounds and flavonoids. The extract exhibited greater cytotoxic activity than that observed in our study, with IC_50_ values of 713.8 µg/mL for MCF-7 cells and 1397 µg/mL for HDF. Despite this difference in potency, both studies reported selective toxicity toward tumor cells.

Regarding the cytotoxic activity reported for essential oils from other plant species, and considering the previously mentioned limitations in their comparison, the study by Oktavianawati et al. is particularly relevant [46]. In their study, the essential oil of *Gonystylus bancanus*, rich in sesquiterpenes and monoterpenes, exhibited cytotoxic activity against MCF-7 and HT-29 cell lines (breast and colorectal adenocarcinomas), with IC_50_ values of 102.34 and 836.46 µg/mL, respectively. Although that study reported greater cytotoxic activity than observed in our study, it provides a valuable basis for future research aimed at obtaining OcEO fractions enriched in potentially bioactive compounds of interest for cancer research. Furthermore, the dose–response behavior observed in the present study supports the presence of bioactive phytochemicals that may contribute to differential cytotoxic effects observed.

Among the major phytochemicals identified in OcEO, in isolation, β-caryophyllene has been reported to inhibit the PI3K/Akt/mTOR signaling pathway in breast and colon adenocarcinoma models, thereby decreasing cell proliferation through cell-cycle arrest at the G1/S phase [47]. Moreover, essential oils rich in germacrene D induce cytochrome c release and subsequently increase the cleaved caspase-3 levels and the Bax/Bcl-2 ratio in cervical and colorectal carcinoma cells [48]. Regarding the activity of the major monoterpene constituent, in isolation, limonene has been shown to interfere with the expression of the oncogenic protein Ras. In addition, it exerts anticancer effects by upregulating genes involved in autophagy, as well as Bax and caspase-3, while downregulating cyclin D1 and Bcl-2 [49,50]. Furthermore, isolated pinenes have been reported to affect tumor cells by modulating the expression of pro- and anti-apoptotic genes, increasing Bax and decreasing Bcl-2 [51,52]. Altogether, these findings provide a solid basis for hypothesizing the potential molecular mechanisms underlying the cytotoxic effects of OcEO.

In summary, the combined effects of β-caryophyllene, germacrene D, limonene, and pinenes may hypothetically contribute to the differential cytotoxic activity of OcEO. Our results support the need for bioassay-guided fractionation studies to isolate and enrich these bioactive fractions, as well as to thoroughly evaluate the molecular pathways underlying the cytotoxic activity of OcEO.

## 3. Materials and Methods

### 3.1. Plant Material and Essential Oil Isolation

Aerial parts (leaves and flowers) of wild plants were collected at 3170 m a. s. l. in Cajas de Loma, Chorocopte, Chañar Province of Ecuador (2° 35′ 1.08″ S, 78° 57′ 0.08″ W) in October 2023. The species was identified, and a voucher specimen was deposited in the herbarium of the Pontificia Universidad Católica del Ecuador (QCAZ-750795). The essential oil was obtained by hydrodistillation. For each extraction, exactly 200 g of fresh plant material was subjected to hydrodistillation using a Clevenger-type apparatus for 3 h. The extraction was performed in three independent replicates. The density of essential oil was measured by the pycnometric method at 20 °C. The resulting essential oil was dried over anhydrous sodium sulfate and stored at 4 °C until analysis.

### 3.2. GC-MS and GC-FID Analysis

GC-MS and GC-FID analyses were performed as previously described by Moncayo-Molina et al. [53]. Briefly, qualitative analysis was carried out by gas chromatography–mass spectrometry (GC-MS) using a Shimadzu QP-2010 Ultra system (Kioto, Japan) equipped with two capillary columns, DB-5ms and DB-Wax (both 30 m in length × 0.25 mm i.d., 0.25 μm film thickness; J & W Scientific, Folsom, CA, USA). Chromatographic separation was carried out using the following temperature program: an initial temperature of 70 °C was held for 2 min, followed by a temperature ramp of 4 °C/min to 250 °C, which was then maintained for 10 min. Both the inlet and MS transfer line were maintained at 250 °C. Compound identification was achieved by comparing linear retention indices (LRI)—calculated using a C8–C24 n-alkane series—and mass spectral fragmentation patterns with those available in reference mass spectral databases (NIST 05, Wiley 6, Adams 2007, NBS 75 k) and an internal library (Flavorlib). When the mass spectra were compared with those in the reference databases, a confidence level ≥ 80% was considered acceptable for positive identification. The essential oil components were quantified using a Hewlett-Packard 6890N Series II gas chromatograph (Agilent, Santa Clara, CA, USA) equipped with a flame ionization detector (GC-FID) and a DB-5ms capillary column (30 m in length × 0.25 mm internal diameter (i. d) × 0.25 μm film thickness; J & W Scientific, Folsom, CA, USA). The oven temperature program, carrier gas flow rate, and injection mode were identical to those described for GC-MS. Mass spectra were acquired in electron ionization (EI) mode at 70 eV, scanning over a mass range of 35–400 m/z. Both the injector and FID ports were maintained at 250 °C. Helium was employed as the carrier gas with a constant flow rate of 1 mL/min. Sample aliquots of 1 μL (diluted to 10% *v*/*v* in diethyl ether) were introduced via split injection (1:20 ratio). The relative abundance of essential oil components was determined by conventional normalization of chromatographic peak areas, assuming an identical response factor for all compounds, a widely accepted approach for comparing complex chemical profiles [54,55]. When no internal standards were used, the results were reported as uncorrected relative GC-FID peak-area percentages. Data are presented as mean ± standard deviation of three independent hydrodistillations, each analyzed in duplicate.

### 3.3. 2,2-Diphenyl-2-picrylhydrazyl (DPPH) Scavenging Activity

Free radical-scavenging activity was assessed using the DPPH assay, following the procedure previously described by Tabart et al. [56]. Stock solutions of the essential oil were prepared in dimethyl sulfoxide (DMSO, Sigma-Aldrich, Milwaukee, WI, USA), and working solutions were prepared at concentrations ranging from 10 to 1000 µL/mL. For the assay, an aliquot of 100 µL of the sample (or standard) was mixed with 2.9 mL of the DPPH reagent solution (100 µM) (Sigma-Aldrich, Milwaukee, WI, USA). Pure DMSO was used as the measurement blank. The mixture was incubated for 30 min in the dark, and absorbance was measured at 517 nm using a Multi-skan FC microplate photometer (Thermo Scientific, Waltham, MA, USA). A calibration curve was constructed using Trolox (Sigma-Aldrich, Burlington, MA, USA) as the reference standard over a concentration range of 0–50 mg/L. Antioxidant capacity was determined using the linear regression equation: x = (y − b)/m, where “x” represents the calculated antioxidant concentration in the sample (expressed as milligrams of Trolox equivalents per gram of fresh sample material, mg TE/g), “y” is the sample’s measured absorbance, “m” is the slope of the calibration curve, and “b” is the *Y*-axis intercept. Only values obtained from calibration curves with an R^2^ > 0.95 (coefficient of determination) were considered valid. Antioxidant activity values, initially expressed as mg TE per µL of OcEO based on the calibration curves, were converted to a fresh plant material basis and expressed as mg TE/g of fresh material (FM). This conversion was performed using the following equation: Antioxidant capacity (mg TE/g FM) = [Antioxidant Capacity (mg TE/µL OcEO)/OcEO density (µg/mL)] × yield (mg OcEO/g FM). Results are presented as mean ± standard deviation of three independent samples, each analyzed in triplicate.

### 3.4. Azino-bis (3-ethylbenzothiazoline-6-sulfonic Acid (ABTS) Assay

The ABTS assay was carried out according to the procedure described by Re et al. [57]. The ABTS•^+^ radical was generated by reacting ABTS (7 mM) (Sigma-Aldrich, Burlington, MA, USA) with potassium persulfate (2.45 mM) (Sigma-Aldrich, Burlington, MA, USA), followed by incubation in the dark at room temperature for 16 h. Once the ABTS•^+^ radical had formed, the solution was diluted with methanol until an absorbance of 0.700 was reached at 734 nm. The essential oil samples were dissolved in DMSO (, Sigma-Aldrich, Milwaukee, WI, USA) to prepare working solutions at concentrations ranging from 10 to 1000 µL/mL. For the reaction, an aliquot of 100 µL of the sample was mixed with 1 mL of the ABTS•+ reagent solution. Pure DMSO was used as the measurement blank. After 10 min incubation, the absorbance was measured at 734 nm using a Multi-skan FC microplate photometer (Thermo Scientific, Waltham, MA, USA). A standard calibration curve was constructed using Trolox over a concentration range of 0–50 mg/L. Antioxidant activity was calculated by linear regression and expressed as milligrams of Trolox equivalents per gram of fresh sample material (mg TE/g), as previously described. Results are presented as mean ± standard deviation of three independent samples, each analyzed in triplicate.

### 3.5. Ferric-Reducing Antioxidant Power (FRAP) Assay

FRAP assay was performed as previously described by Benzie and Strain [58]. Briefly, essential oil samples were diluted in DMSO (Sigma-Aldrich, Milwaukee, WI, USA) to prepare working solutions at concentrations ranging from 10 to 1000 µL/mL. The working FRAP (Sigma-Aldrich, Burlington, MA, USA) solution, consisting of300 mM acetate buffer (pH 3.6), 10 mM TPTZ (2,4,6-tripyridyl-s-triazine) solution in 40 mM HCl, and 20 mM FeCl_3_·6H_2_O (ferric chloride hexahydrate) solution mixed at a 10:1:1 (v/v/v) ratio, was freshly prepared before the analysis. For the assay, an aliquot of 10 µL of the sample was mixed with 100 µL of the FRAP reagent solution in 96-well microplates. Pure DMSO was used as the measurement blank. The reaction mixture was incubated for 4 min at room temperature, and the absorbance was measured at 593 nm using a Multi-skan FC microplate photometer (Thermo Scientific, Waltham, MA, USA). A standard calibration curve was constructed using Trolox over a concentration range of 0–50 mg/L. Ferric reducing antioxidant power was calculated using linear regression and expressed as milligrams of Trolox equivalents per gram of fresh sample material (mg TE/g), as previously described. Results are presented as mean ± standard deviation of three independent samples, each analyzed in triplicate.

### 3.6. Antimicrobial Activity Determination

The microbial strains, obtained from the American Type Culture Collection (ATCC; Manassas, VA, USA), were *Klebsiella pneumoniae* (ATCC 13883), *Acinetobacter baumannii* (ATCC 19606), and the pathogenic yeast *Candida albicans* (ATCC 10231). OcEO samples were diluted in Mueller-Hinton broth (MHB) containing 1% DMSO to ensure essential oil solubilization. Bacterial strains were cultured on tryptic soy agar (TSA) at 37 °C for 24 h, whereas *C. albicans* was cultured on Sabouraud agar at 28 °C for 48 h before testing. Antimicrobial activity was evaluated using the agar disc diffusion assay and by determining the minimum inhibitory concentration (MIC), following CLSI guidelines [59]. The MIC was determined by the broth microdilution assay in 96-well U-bottom micro-titer plates, using a final concentration range of 0.31–40 mg/mL. Each well was inoculated to a final concentration of 5 × 10^5^ CFU/mL. MIC endpoints were determined instrumentally using a Multi-skan FC microplate photometer (Thermo Scientific, Waltham, MA, USA) by measuring the optical density at 600 nm. The MIC was defined as the lowest concentration that completely inhibited bacterial growth after 24 h of incubation. The zone of inhibition (ZOI) was calculated by subtracting the disc diameter from the total clear zone diameter. A volume of 20 μL of pure OcEO was used to impregnate the paper disks (6 mm in diameter); ciprofloxacin (30 μg/20 μL) and ketoconazole (33 μg/20 μL) were used as positive controls, and 1% DMSO in MHB (20 μL) served as the negative control. All experiments were performed using three independent samples in triplicate. Results are expressed as mean values ± standard deviation.

### 3.7. Cell Lines and Culture Conditions

The human breast adenocarcinoma cell line MCF-7 (ATCC, Manassas, VA, USA), the human colorectal cancer cell line HCT-116 (ECACC, Saint Louis, MO, USA), and the human normal lung fibroblast cell line MRC-5 (ABAC, Cordoba, Argentina) were cultured in DMEM (Merck, Darmstadt, Germany) supplemented with 10% fetal bovine serum (Internegocios, Cordoba, Argentina),100 IU/mL of penicillin and 100 µg/mL of streptomycin (GIBCO, NY, USA). Cell cultures were maintained at 37 °C in a humidified incubator with 5% CO_2_ (Thermo Fisher Scientific Inc., Waltham, MA, USA).

### 3.8. 3-(4,5-Dimethylthiazol-2-yl)-2,5-diphenyltetrazolium bromide (MTT) Cytotoxicity Assay

OcEO-induced cytotoxicity was determined using the MTT (3-(4,5-dimethylthiazol-2-yl)-2,5-diphenyl tetrazolium bromide) assay (Sigma, Saint Louis, MO, USA) as previously reported [60]. Briefly, cells were seeded at a density of 5 × 10^3^ cells per well in 96-well plates containing 100 µL of culture medium. After 24 h, the medium was aspirated and replaced with fresh medium containing 1% DMSO to ensure adequate solubilization of the essential oil, along with OcEO at the indicated concentrations (0.28–89.4 mg/mL). The plates were then incubated for 48 h. Doxorubicin (Microsules, CABA, Argentina) at 0.1 µM was used as the positive and inter-assay control, whereas 1% DMSO served as the negative control. Optical density was measured at 570 nm using a Thermo Scientific Multiscan microplate reader (Thermo Scientific, Waltham, MA, USA). The optical density of untreated control cells was considered 100% proliferation. Assays were performed using three independent samples in triplicate. The IC_50_ values were determined by sigmoidal dose–response analysis using GraphPad Prism 9.0 software (Graph Pad Software, Inc., San Diego, CA, USA); fits were considered acceptable when R^2^ ≥ 0.8. The Selectivity Cytotoxicity Index (SCI) was calculated as the ratio of the IC_50_ value for the non-tumor MRC-5 cell line to that of the corresponding tumor cell line (IC_50_ MRC-5/IC_50_ tumor cells).

### 3.9. Statistical Analysis

Statistical analyses were performed using GraphPad Prism 9.0 software (GraphPad Software Inc., San Diego, CA, USA). Data are expressed as mean ± standard deviation (SD). Differences between groups were assessed using Student’s t test or one-way ANOVA, as appropriate. Prior to performing the analysis of variance, the underlying assumptions were verified: data normality was assessed using the Shapiro–Wilk test, and homogeneity of variances was validated through Bartlett’s test (*p* > 0.05). When significant differences were detected by ANOVA, means were subsequently compared using Dunnett’s post hoc test. Statistical significance was set at *p* < 0.05. All in vitro experiments were performed using three independent biological replicates, corresponding to samples obtained from separate hydrodistillation, with each replicate analyzed in triplicate as technical replicates.

## 4. Conclusions

This study provides the first comprehensive characterization of the essential oil of *O. corniculata* from the Ecuadorian highlands, revealing a complex chemical profile comprising 17 identified and 7 tentatively identified phytochemicals, predominantly sesquiterpenes and monoterpenes. OcEO exhibited complementary chemical antioxidant activity in the DPPH, ABTS, and FRAP assays, together with antimicrobial activity against Gram-negative bacteria and *C. albicans*. Moreover, its cytotoxic effects were more pronounced in HCT-116 and MCF-7 tumor cells than in MRC-5 normal fibroblasts, with selective cytotoxicity indices exceeding the threshold reported in the literature for both tumor cell lines. The combined presence of major constituents such as β-caryophyllene, germacrene D, limonene, and pinenes may hypothetically contribute to these biological effects, although their individual contributions and potential synergistic interactions remain to be established. Overall, these findings provide preliminary evidence supporting the bioactive and biotechnological potential of OcEO. Further validation is required to support the bioprospecting of this Ecuadorian species and encourage future research involving bioassay-guided fractionation and mechanistic studies.

## Figures and Tables

**Figure 1:**
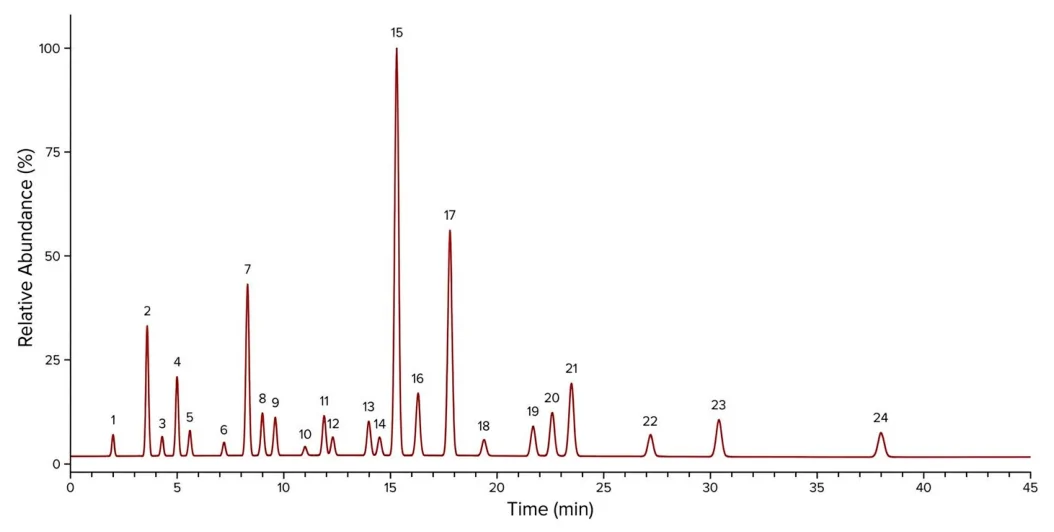
Total ion chromatogram of OcEO obtained on a DB-5ms column.

**Figure 2:**
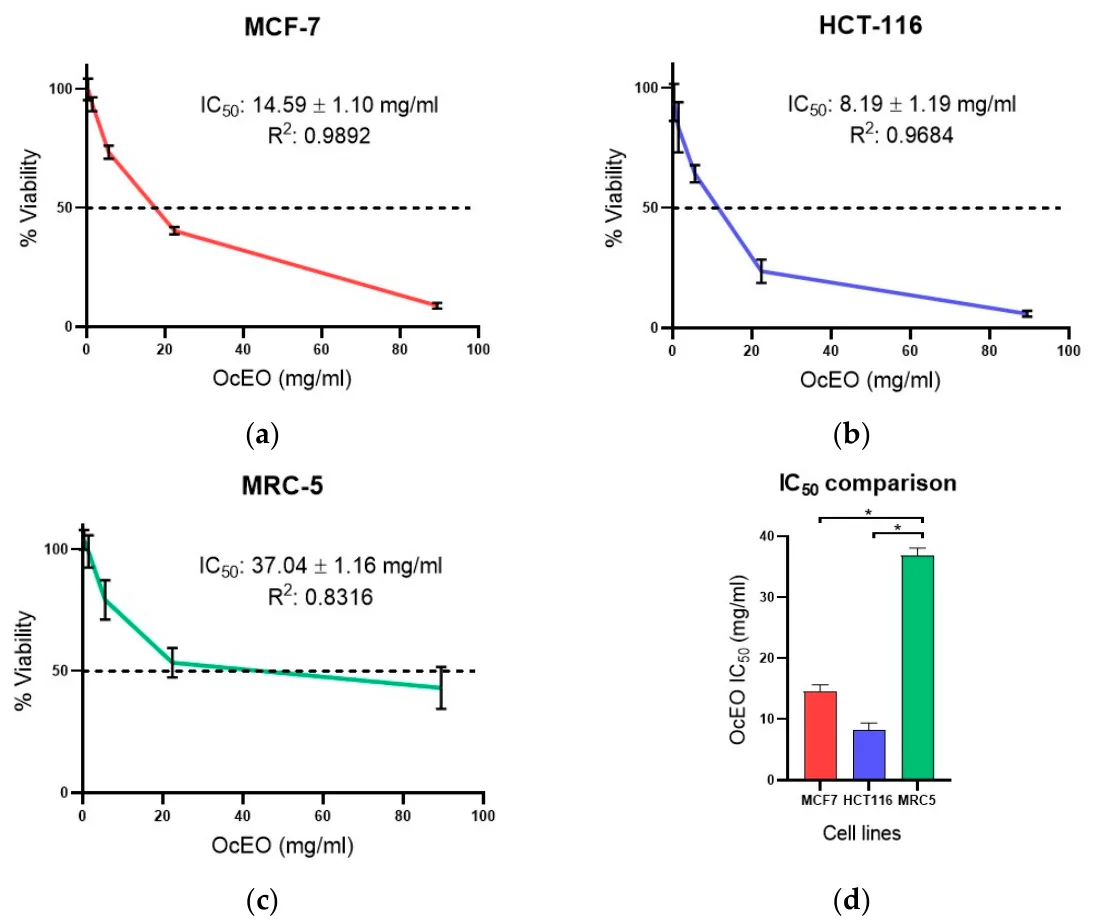
Cytotoxicity of OcEO against tumors and non-tumor human cell lines. Dose–response curves for (**a**) MCF-7 (human breast adenocarcinoma), (**b**) HCT-116 (human colorectal carcinoma), and (**c**) MRC-5 (human normal lung fibroblasts). (**d**) IC_50_ (concentration required to inhibit 50% of cell proliferation) comparison among the studied cell lines. R^2^: Goodness of fit for the sigmoidal dose–response analysis. Results are presented as mean ± SD from three independent experiments performed in triplicate. *: indicates significant differences between MRC5 and MCF-7, and between MRC5 and HCT-116 (ANOVA, followed by Dunnett’s post hoc test, *p* < 0.0001).

**Table 1:** Metabolite profile of OcEO.

Peak	Compound	RI-5^e^	RI-5^s^	RI-W^e^	RI-W^s^	% RA
1	Hexanaldehyde *	803	801	1083	1084	0.8 ± 0.02
2	α-Pinene	939	939	1029	1026	5.27 ± 0.15
3	Sabinene	976	975	1118	1115	0.79 ± 0.02
4	β-Pinene	979	979	1121	1125	3.4 ± 0.09
5	Myrcene	991	989	1165	1160	1.07 ± 0.03
6	*p*-Cymene	1024	1025	1262	1265	0.59 ± 0.02
7	Limonene	1031	1029	1198	1195	8.36 ± 0.24
8	1,8-Cineole	1033	1031	1212	1212	2.1 ± 0.06
9	γ-Terpinene	1062	1060	1257	1254	1.92 ± 0.05
10	Fenchone *	1122	1088	1400	1407	0.46 ± 0.01
11	Terpinen-4-ol	1177	1177	1601	1606	2.18 ± 0.06
12	α-Terpineol *	1189	1192	1675	1677	1.02 ± 0.03
13	Thymol	1298	1290	2149	2153	2.01 ± 0.05
14	Carvacrol	1299	1299	2219	2222	1.07 ± 0.02
15	β-Caryophyllene	1417	1419	1596	1598	24.71 ± 0.71
16	α-Humulene	1452	1455	1675	1670	3.88 ± 0.11
17	Germacrene D	1480	1485	1697	1693	14.62 ± 0.39
18	β-Farnesene	1505	1491	1745	1747	1.07 ±0.03
19	Nerolidol	1563	1533	2005	2008	2.1 ± 0.06
20	Spathulenol *	1577	1575	2136	2140	3.17 ± 0.08
21	Caryophyllene oxide	1583	1583	1986	1983	5.41 ± 0.14
22	Hexadecanoic acid **	---	1960	---	2910	1.72 ± 0.04
23	Phytol **	---	2110	---	2617	3.13 ± 0.09
24	Squalene **	---	2814	---	2865	2.36 ± 0.09

RI-5^e^: mean of experimental linear retention indices on a DB-5ms column. RI-5^s^: linear retention indices from standards or the literature on a DB-5 ms column. RI-W^e^: mean of experimental linear retention indices on a DB-Wax column. RI-5W^s^: linear retention indices from standards or the literature on a DB-Wax column. %RA: Percent relative abundance. * Tentatively identified compounds based on comparison with literature data (NIST 05, Wiley 6, Adams 2007, and NBS 75 k databases). ** Hexadecanoic acid, phytol and squalene were identified based on high-probability matches of their mass spectra with the aforementioned reference databases. For compounds marked with * and **, relative abundance percentages should be considered uncorrected relative peak-area percentages. Literature values on DB-5ms and DB-Wax columns were adopted for reporting purposes.

**Table 2:** In vitro antioxidant capacity of OcEO.

Antioxidant Assay	mg TE/g
DPPH radical-scavenging ability	15.87 ± 0.12
ABTS radical-scavenging ability	17.97 ± 0.09
FRAP ferric-reducing antioxidant power	12.39 ± 0.10

Values are expressed as mean ± standard deviation.

**Table 3:** Antimicrobial activity of OcEO.

Microbial Strains	OcEO		CPX		KTC	
	MIC (mg/mL)	ZOI (mm)	MIC (mg/mL)	ZOI (mm)	MIC (mg/mL)	ZOI (mm)
*K. pneumoniae*	0.56 ± 0.05 ^a^	11.41 ± 0.9 ^d^	0.27 ± 0.01 ^a^	31.7 ± 0.36 ^d^	----------	----------
*A. baumanii*	4.47 ± 0.94 ^b^	8.85 ± 0.6 ^e^	0.29 ± 0.01 ^b^	31.3 ± 0.11 ^e^	----------	----------
*C. albicans*	2.24 ± 0.62 ^c^	14.40 ± 1.1 ^f^	----------	----------	0.13 ± 0.01 ^c^	21.4 ± 0.41 ^f^

CPX: Ciprofloxacin; KTC: Ketoconazole. Superscript letters indicate statistical differences (Student’s t test): a: *p* = 0.0006; b: *p* = 0.0015; c: *p* = 0.0041; d and e: *p* < 0.0001; f: *p* = 0.0005.

**Table 4:** Cytotoxic activity of OcEO.

OcEO (mg/mL)	MCF-7 (% viab)		HCT-116 (% viab)		MRC-5 (% viab)	
	Mean	SD	Mean	SD	Mean	SD
*0.28*	99.80	4.48	93.94	7.72	103.83	4.04
*1.40*	93.54	2.93	83.57	10.48	99.05	6.62
*5.59*	73.35	2.82	64.17	3. 59	79.14	8.09
*22.35*	40.34	1.55	23.61	4.86	53.43	6.10
*89.40*	8.86	1.25	5.90	1.20	43.07	8.67

MCF-7: Human breast adenocarcinoma. HCT-116: Human colorectal carcinoma. MRC-5: Human normal lung fibroblasts. % Viab: percentage of viability obtained via the MTT assay. Results represent three independent experiments performed in triplicate.

## Data Availability

After publication, data will be placed at the “Repositorio de datos del CONICET” (https://ri.conicet.gov.ar/).
